# Non-Volatile and Volatile Bioactives of *Salvia officinalis* L., *Thymus serpyllum* L. and *Laurus nobilis* L. Extracts with Potential Use in the Development of Functional Beverages

**DOI:** 10.3390/antiox11061140

**Published:** 2022-06-10

**Authors:** Ivanka Maleš, Verica Dragović-Uzelac, Igor Jerković, Zoran Zorić, Sandra Pedisić, Maja Repajić, Ivona Elez Garofulić, Ana Dobrinčić

**Affiliations:** 1 Department of Pharmacy, The School of Medicine, University of Split, 21000 Split, Croatia; ivanka.males@mefst.hr; 2 Faculty of Food Technology and Biotechnology, University of Zagreb, 10000 Zagreb, Croatia; zzoric@pbf.hr (Z.Z.); spedisic@pbf.hr (S.P.); maja.repajic@pbf.unizg.hr (M.R.); ielez@pbf.hr (I.E.G.); 3 Department of Organic Chemistry, Faculty of Chemistry and Technology, University of Split, 21000 Split, Croatia; igor@ktf-split.hr

**Keywords:** herbal extracts, functional beverages, antioxidant capacity, non-volatiles, volatiles, *Salvia officinalis* L., *Thymus serpyllum* L., *Laurus nobilis* L.

## Abstract

Functional beverages based on herbal extracts are highly demanded products due to the presence of bioactives with promising health benefits and interesting and characteristic sensory properties. Mediterranean medicinal and aromatic herbs contain a wide range of bioactives (non-volatile polyphenols, volatile terpenes) that are important constituents of herbal extracts and essential oils. The antioxidant capacity and potential health benefits of these bioactives could be associated with their synergistic effects. Therefore, this study aimed to characterize the non-volatile and volatile bioactives of sage *(Salvia officinalis* L.), wild thyme (*Thymus serpyllum* L.) and laurel (*Laurus nobilis* L.) aqueous extracts and their two- and three-component mixtures as well as their antioxidant capacity. The content of total phenols, flavonoids, hydroxycinnamic acids and flavonols was determined spectrophotometrically. Individual polyphenols were analyzed by LC-MS/MS, the volatiles were analyzed by HS-SPME/GC-MS, and the antioxidant capacity was analyzed by ORAC and DPPH assays. The results showed that aqueous extracts of all examined herbs and their mixtures contained a high content of phenolic compounds ranging from 0.97 to 2.79 g L^−1^ of the sample, among which the most common were flavonols. At the same time, mono- and sesquiterpenes were the main volatiles. All extracts showed high antioxidant capacity, especially *L. nobilis* (781.62 ± 5.19 μmol TE mL^−1^ of the sample in the DPPH assay; 1896.10 ± 8.77 μmol TE mL^−1^ of the sample in the ORAC assay) and the two-component mixture of *L. nobilis* and *T. serpyllum* (679.12 ± 5.19 μmol TE mL^−1^ in the DPPH assay; 1913.38 ± 8.77 μmol TE mL^−1^ in the ORAC assay). Mixtures of herbal extracts have been shown to possess additive or synergistic effects, consequently contributing to higher antioxidant capacity. Therefore, two-component mixtures of herbal extracts showed promising potential for the production of functional beverages.

## 1. Introduction

Medicinal and aromatic herbs represent a valuable source of phytochemicals that have strong biological activities and health benefits [1]. Extracts of medicinal and aromatic herbs are often added to functional food products, thus becoming desirable ingredients for functional beverages to improve overall health conditions [2]. Functional beverages based on herbal extracts contain a wide range of bioactive molecules responsible for potential antioxidant, anti-inflammatory, anticholesterolemic, antitumor, and other beneficial properties [3,4,5,6]. The selection of medicinal and aromatic herbs and the combination of several plant species in the production of herbal extracts is performed depending on the composition and content of bioactive molecules, potential synergistic effects, and the type of functional beverage to obtain high-quality beverages with increased antioxidant activity and other effective properties [5,6]. Mediterranean herbs such as sage, wild thyme, and laurel contain a specific composition of bioactive molecules (non-volatile polyphenols, volatile terpenes) that could potentially contribute to the functional and sensory properties of target products. A wide range of polyphenols found in these herbs includes various flavonoids and phenolic acids [7,8,9,10,11], which have high solubility in water extracts. In contrast, during the production of herbal extracts, only a small fraction of volatiles (mainly from the essential oils) passes into aqueous extracts [12,13,14]. Their contribution to the herbal extract biopotential is minor, but even at very low concentrations, they may contribute to the sensory, especially the aromatic, properties of functional beverages.

Polyphenols are the most abundant non-volatile bioactives in sage, wild thyme, and laurel that possess antioxidant, antimicrobial, anticarcinogenic, antifungal, antidiabetic, and anti-inflammatory effects, and some of these effects can often be enhanced due to synergistic effects among structurally diverse polyphenols [9,15,16,17,18,19,20,21,22,23]. The synergism of mixtures of major or minor phenolics affects different pathways of the disease and contributes to faster and more effective healing [24]. In addition, a synergistic effect between some volatile constituents has also been observed, which is especially important in the production of functional beverages as they can inhibit or reduce the natural oxidation process of the products [25,26,27,28].

Although sage, wild thyme, and laurel extracts are recognized as rich sources of polyphenols and specific volatile compounds, there is still insufficient data and necessary knowledge about their potential synergistic effects and their application in the preparation of functional beverages. Therefore, the aims of this study (first-time report) were to (a) characterize the main bioactive molecules of sage, wild thyme and laurel; (b) determine the biopotential of one-, two- and three-component extracts of selected herbs and (c) define the formulations with the highest antioxidant capacity (DPPH radical scavenging and oxygen radical absorbance capacity) for potential application in the enrichment or development of a new functional beverage.

## 2. Materials and Methods

### 2.1. Chemicals

Formic acid and acetonitrile were HPLC grade, purchased from BDH Prolabo, VWR (Lutterworth, England). Distilled water was Milli-Q quality (Millipore Corp., Bedford, NY, USA). Fluorescein sodium salt was obtained from Honeywell Riedel-de-Haën (Bucharest, Romania), Trolox (6-hydroxy-2,5,7,8-tetramethylchroman-2-carboxylic acid) was obtained from Acros Organics (Thermo Fisher Scientific, Geel, Belgium), and 2,20-azobis(2-amidinopropane) hydrochloride, DPPH-2,2′-diphenyl-1-picrylhydrazyl and aluminium chloride were purchased from Sigma-Aldrich (Steinheim, Germany). A Folin–Ciocalteu reagent was obtained from Fisher Chemical. Ethanol and sodium carbonate were obtained from Gram-mol Company (Zagreb, Croatia). Hydrochloric acid was purchased from Carlo Erba Reagents S.r.l. (Val-de-Reuil, France), and potassium acetate was purchased from VWRChemicals (Radnor, PA, USA). Methanol (HPLC Chromasolv) was purchased from Riedel-de Haën GmbH & Co. (Seelze, Germany).

A commercial phenolic compound of authentic standards of gallic acid, caffeic acid, ferulic acid, chlorogenic acid, *p*-coumaric acid, kaempferol-3-rutinoside and quercetin-3-glucoside was purchased from Sigma-Aldrich (Steinheim, Germany). Catechin, epicatechin, epigallocatechin gallate, epicatechin gallate, procyanidin B1, apigenin, and luteolin were purchased from Extrasynthese (Genay, France), and quercetin-3-rutinoside was purchased from Acros Organics (Thermo Fisher Scientific, Geel, Belgium).

### 2.2. Herbal Material

The samples of sage *(Salvia officinalis* L.) (*S*), wild thyme (*Thymus serpyllum* L.) (*WT*) and laurel (*Laurus nobilis* L.) (*L*) leaves were purchased from Suban Ltd. (Strmec Samoborski, Croatia), a certified collector and producer of medicinal and aromatic herbs. The herbs were harvested in 2020, packaged in their original packages (paper bags) and stored in a dark and dry place. Before the extraction, they were ground using an electric grinding machine (WSG30, Waring Commercial, Torrington, CT, USA).

### 2.3. Herbal Extract Preparation

Ground dried sage, wild thyme and bay leaves (30 g) were extracted with distilled water (200 mL) at 60 °C for 30 min on a horizontal water bath shaker (Memmert WB14, SV1422, Schwabach, Germany). The extracts were filtered through Whatman no. 40 filter paper (Whatman International Ltd., Kent, UK) and filled to a constant volume (200 mL). Two-component and three-component mixtures were prepared in different ratios according to Table 1. Prepared samples were used for the determination of polyphenols and antioxidant capacity as well as for headspace solid-phase microextraction (HS-SPME). All samples were prepared in duplicate and stored at 4 °C (no longer than 7 days).

### 2.4. Determination of Total Phenol Content

The total phenol content (TPC) of the extracts was determined using a spectrophotometric method based on the color reaction of phenols with Folin–Ciocalteu’s reagent [29]. The reaction mixture was prepared by mixing 100 µL of the herbal extract, 200 µL Folin–Ciocalteu’s reagent (undiluted), 2 mL of distilled water and 1 mL of 20% sodium carbonate solution, which was added after 3 min. The mixtures were mixed in a vortex and kept at 50 °C in a water bath for 25 min. The optical density of the solution (absorbance) was measured using a spectrophotometer (UV–1600PC, VWR International, Radnor, PA, USA) at 765 nm. The measurements were carried out in duplicate, and distilled water was used in the reaction as a blank. TPC was calculated according to the gallic acid standard calibration curve (y = 0.0035x, R^2^ = 0.9995), and the results are expressed as as mean values ± standard deviation of g L^−1^ of the sample (*n* = 2 replicates).

### 2.5. Determination of Total Flavonoid Content

The spectrophotometric method to determine total flavonoid content (TFC) in herbal extracts is based on the color reaction of flavonoids with aluminium chloride and potassium acetate [30]. The reaction mixture was prepared by mixing 0.5 mL of the herbal extract, 1.5 mL of 96% ethanol, 0.1 mL of 10% aluminum chloride, 0.1 mL of 1 M potassium acetate and 2.8 mL of distilled water. The measurements were carried out in duplicate, and the blank was prepared using the same protocol with distilled water instead of the herbal extract and 10% aluminium chloride. Prepared mixtures were kept at room temperature for 30 min, after which the absorbance was measured at 415 nm. The concentration of the TFC was calculated using a calibration curve for quercetin (y = 0.0069x + 0.0002, R^2^ = 0.9977), and the results were expressed as mean values ± standard deviation of g L^−1^ of the sample (*n* = 2 replicates).

### 2.6. Determination of Total Hydroxycinnamic Acid and Flavonol Content

Total hydroxycinnamic acid (THCA) and flavonol content (TFLC) were determined according to the method conducted by Howard et al. [31]. The reaction mixture was prepared by mixing 250 µL of the extracts, 250 μL of 1 g L^−1^ hydrochloric acid (mixed with 96% ethanol) and 4.55 mL of 2 g L^−1^ hydrochloric acid (mixed with distilled water). Afterward, the absorbance was measured at 320 and 360 nm in duplicate. The blank was prepared in the same way, with distilled water instead of the herbal extract. Quantification of the THCA was made with the caffeic acid calibration curve (y = 0.0047x + 0.0231, R^2^ = 0.9998), while the quantification of the TFLC was made with the quercetin calibration curve (y = 0.0031x, R^2^ = 0.9975). The results are expressed as mean values ± standard deviation of g L^−1^ of the sample (*n* = 2 replicates).

### 2.7. LC-MS/MS Chromatography

Separation of targeted phenolic compounds was performed by an ultra performance liquid chromatography (UPLC) system (Agilent series 1290 RRLC instrument, Agilent, Santa Clara, CA, USA) using a Fortis C18 column 100 × 2.1 mm with 1.7 µm particle size (Fortis Technologies Ltd., Neston, UK). The eluent compositions and the gradient conditions were previously described by Elez Garofulić et al. [32]. The identification and quantification of phenolic compounds were performed on a 64,300 QqQ mass spectrometer (Agilent) in both ionization modes. Briefly, the analytes were ionized by the ESI ion source with N_2_ as a desolvation and collision gas at a flow rate of 11 L h^−1^ and temperature of 300 °C. The nebulizer pressure was set at 40 psi, and the capillary voltage was set at +4 and −3.5 kV. Agilent MassHunter Workstation Software (v. B.04.01) was used for instrument control and data analysis. Quantitative determination was carried out using the calibration curves of the standards as follows: (a) kaempferol rutinoside, kaempferol hexoside, kaempferol deoxyhexoside and kaempferol pentoside were calculated according to kaempferol-3-glucoside; (b) isorhamnetin hexoside, quercetin rhamnoside, quercetin pentoside were calculated according to quercetin-3-glucoside; (c) apigenin-6-C-(*O*-deoxyhexosyl)-hexoside was calculated according to apigenin; (d) luteolin-6-C-glucoside was calculated according to luteolin; (e) epicatechin was expressed as catechin equivalents; (f) 3,4-dihidrobenzoic acid hexoside was calculated as protocatechuic acid; and (g) *p*-hydroxybenzoic acid was calculated as gallic acid. All analyses were performed in duplicate, and concentrations of analyzed compounds are expressed as as mean values ± standard deviation of mg L^−1^ of the sample (*n* = 2 replicates).

### 2.8. DPPH Radical Scavenging Activity

The spectrophotometric method was used to measure the ability of the extracts to scavenge the DPPH radicals according to the previously defined method [29]. The herbal extracts (0.75 mL) were mixed with the DPPH solution (0.2 mM in methanol) (1.5 mL), shaken on a vortex mixer and kept in the dark at room temperature for 30 min. The decrease in absorbance was measured at 517 nm in duplicate, and methanol was used as a blank. A Trolox calibration curve (y = –0.008x + 1.3476, R^2^ = 0.9948) was used, and the results are expressed as as mean values ± standard deviation of μmol Trolox equivalent (TE) per mL of sample (*n* = 2 replicates).

### 2.9. Oxygen Radical Absorbance Capacity (ORAC) Assay

The antioxidant capacity of the extracts was assessed by the oxygen radical absorbance capacity (ORAC) assay according to the study of Elez Garofulić et al. [33]. The ORAC procedure used an automated plate reader (BMG LABTECH, Offenburg, Germany) with 96-well plates, and the data were analyzed by MARS 2.0 software. The 2,2′-azobis(2-methylpropionamidine) dihydrochloride (AAPH), fluorescein solution and different dilutions of 6-hydroxy-2,5,7,8-tetramethylchroman-2-carboxylic acid (Trolox) were prepared in 75 μM phosphate buffer (pH 7.4). Briefly, diluted samples were added to a 96-well black plate containing a fluorescein solution (70.3 nM) and incubated for 30 min at 37 °C. After the incubation, fluorescence measurements (the excitation at 485 nm and emission at 520 nm) were taken every 90 sec. to determine the background signal. After three cycles, AAPH (240 mM) was injected into each well to initiate the peroxyl radical generation. Different dilutions of Trolox were used on each plate as the reference standard. The fluorescence intensity was monitored over a total measurement period of 120 min. The measurements were performed in duplicate, and the results are expressed as as mean values ± standard deviation of μmol TE per mL of sample (*n* = 2 replicates).

### 2.10. Headspace Solid-Phase Microextraction and GC-MS (HS-SPME/GC-MS)

HS-SPME was conducted using a manual SPME holder and polydimethylsiloxane/divinylbenzene (PDMS/DVB) fibre purchased from Supelco Co. (Bellefonte, PA, USA). The fibre was conditioned using the instructions by Supelco Co. Samples of the herbal extracts and their mixtures were placed separately in 5 mL glass vials immediately after extraction (2 mL) and closed hermetically with PTFE/silicone septa. The vials were kept in a water bath at 60 °C during equilibration (15 min) and HS-SPME (45 min) and were partially submerged so that the liquid phase of the sample was below the water level. The extraction was conducted under constant stirring (1000 rpm) with a magnetic stirrer. An SPME fibre was withdrawn into the needle, removed from the vial, and inserted into the injector (250 °C) of a gas chromatograph with a mass spectrometer (GC-MS). The extracted volatiles were thermally desorbed directly to the GC column after 6 min. All extractions (HS-SPME) were performed in duplicate (*n* = 2 replicates).

GC-MS analyses were performed on a gas chromatograph model 7890A (Agilent Technologies, Palo Alto, CA, USA) and equipped with an HP-5MS capillary column (5% phenyl-methylpolysiloxane, Agilent J and W; 30 m × 0.25 mm i.d., coating thickness 0.25 μm) and a mass selective detector (MSD) model 5977E (Agilent Technologies, Palo Alto, CA, USA). The carrier gas was helium (He 1.0 mL min^−1^). The oven temperature was set at 70 °C for 2 min; then, the temperature was increased from 70 to 200 °C (3 °C min^−1^) and held at 200 °C for 15 min. The MSD (EI mode) was used at 70 eV with a 30–300 amu mass range. The compounds’ identification was based on the retention indices (RIs) determined relative to *n*-alkanes’ (C_9_–C_25_) retention times and their comparison with data in the literature (National Institute of Standards and Technology), as well as by their mass spectra compared with the spectra from Wiley 9 (Wiley, New York, NY, USA) and NIST 17 (D-Gaithersburg) mass spectral libraries. The results are expressed as a percentage composition as mean values ± standard deviation (*n* = 2 replicates).

### 2.11. Statistical Analysis

All extractions and measurements were performed in duplicate. The results were expressed as mean values ± standard deviation, analyzed for statistical significance at *p* ≤ 0.05, using the STATISTICA 8.0 software (StatSoft Inc., Tulsa, OK, USA). Continuous variables were analyzed by one-way analysis of variance (ANOVA), and marginal means were compared with Tukey’s HSD multiple comparison test. Dependent variables were TPC, TFC, THAC, TFLC, DPPH, ORAC and each of the polyphenol compounds detected by HPLC.

## 3. Results and Discussion

### 3.1. Determination of Total Phenol, Flavonoid, Hydroxycinnamic Acid and Flavonol Content

In this study, *S*, *WT* and *L* extracts and their mixtures were analysed for TPC, TFC, THCA and TFLC, and the results are shown in Table 2. The content of THCA and TFLC did not differ significantly (*p* ≥ 0.05) between the herbal extracts and their mixtures, while TPC and TFC values were significantly different (*p* ≤ 0.05).

The TPC for *S* extract content (2.49 g L^−1^ of the sample or 27.05 mg of gallic acid equivalents (GAE) g^−1^ of dry herb) was higher than the 9.15 mg GAE g^−1^ in supercritical fluid sage leaf extract reported by Pavić et al. [34], higher than 17.1 mg GAE g^−1^ in sage methanol-acetone extract determined by Francik et al. [35], and higher than methanolic sage extract in research conducted by Sytar et al. [36] and Hamrouni-Sellami et al. [37], reporting 2.23 mg GAE g^−1^ and 2.337 mg GAE g^−1^, respectively. Similar results (25.58 mg GAE g^−1^) for the hydro-methanolic extract were reported by Doymaz & Karasu [38]. The TFC content for *S* extract (0.62 g L^−1^ or 6.91 mg of quercetin equivalents (QE) g^−1^ of dry herb) was higher than the 0.923 mg of QE g^−1^ determined by Hamrouni-Sellami et al. [37] and similar to the 5 mg QE g^−1^ reported by Sytar et al. [36]. As for *WT* extract, the TPC content was 2.79 g L^−1^ or 15.05 mg GAE g^−1^. This value is higher than the 12.63 mg catechol equivalent (CE) g ^−1^ in methanolic extract determined by Goyal et al. [39] and similar to the values (15.06 mg GAE g^−1^) for thyme flower methanolic extracts reported by Jabri Karoui et al. [40] and the 15.53 mg caffeic acid equivalents (CAE) g^−1^ for an aqueous decoction of *Thymus* x *citriodorus* L. in the study conducted by Taghouti et al. [41]. The TFC for *WT* extract in the present study was 3.17 mg QE g^−1^, which is higher than TFC (ranging from 1.412 to 2.076 mg QE g^−1^) in other *Thymus* species, such as *Thymus trautvetteri* extracts in different solvents [42] and *Thymus vulgaris* L. methanolic extract (1.71 mg QE g^−1^) [43]. Regarding the *L* extract, the TPC was 7.19 mg GAE g^−1.^ This value is higher than the 2.34 mg GAE g^−1^ in an ethanolic extract in research conducted by Muñiz-Márquez et al. [44], higher than the 2.272 mg GAE g^−1^ in the aqueous extract reported by Kashkouli et al. [45], higher than in leaf extracts in different solvents ranging from 0.477 to 0.797 mg GAE g^−1^ according to Tometri et al. [46] and higher than the 4.04 mg GAE g^−1^ in phosphate buffer (75 mM, pH 7.0) extract reported by Zheng and Wang [47]. The TFC for *L* extract was 0.82 mg QE g^−1^, which is higher than the leaf extracts in different solvents ranging from 0.193 to 0.399 mg CE g^−1^ recorded in research by Tometri et al. [46].

The current study results for one-component herbal extracts showed the highest TPC in the *WT* extract, so it was expected that two-component mixtures with *WT* would also have high TPC. Furthermore, low TPC in *L* is reflected in all two- or three-component mixtures, with *L* as the dominant extract showing a lower amount of TPC. The values were higher in two-component mixtures where *L* was not predominant, such as in the *WTL31* (2.33 g L^−1^) and *SL31* mixtures (2.04 g L^−1^), due to high TPC in the individual extracts of *S* and *WT*. However, the highest TPC was determined in the *WTS31* mixture (2.51 g L^−1^). Malongane et al. [48] also reported TPC in two-component herbal teas. They studied the TPC of honeybush (*Cyclopia species*), rooibos (*Aspalathus linearis* (Burm.f.) R.Dahlgren) and special tea (*Monsonia burkeana* L.) in combination with bush tea (*Athrixia phylicoides* Ker Gawl.). The results showed that the special tea contained the highest TPC of all one-component herbal teas (1.10 mg GAE g^−1^ of the dry sample). Still, this content was higher in the mixture with bush tea, where the ratio of the herbals was combined as 75% of special tea and 25% of bush tea (1.44 mg GAE g^−1^ of the dry sample).

The current study results showed that three-component mixtures contained lower TPC than two-component mixtures. The highest TPC was determined in the *WTSL221* mixture (1.45 g L^−1^), which was expected considering that this mixture had a higher amount of *S* and *WT*. However, this value was almost twice lower than the value of the *WT* extract. A similar effect of a combination of more than two herbal extracts was obtained by Studzińska-Sroka et al. [49]. They reported TPC in mulberry (*Morus alba* L.) leaves and mixtures of mulberry leaves with other herbs, made as two-, four-, five-, and six-component herbal tea blends, where the two-component herbal mixture composed of mulberry leaves (70%) combined with cinnamon (*Cinnamomum verum* J.Presl) bark (30%) had the highest content of polyphenols (16.06 mg GAE g^−1^ of the herbal blend). On the contrary, Cheminet et al. [50] reported on two-, three-, four- and five-component decoctions containing yerba mate (*Ilex paraguariensis* A.St.-Hil.) of different commercial brands combined with peppermint (*Mentha x piperita* L.), pennyroyal (*Mentha pulegium* L.), peperina (*Minthostachys verticillata* (Griseb.) Epling), boldo (*Peumus boldus* Molina), melissa (*Melissa officinalis* L.) and lemon verbena (*Aloysia citrodora* Paláu). Their study showed that the five-component mixture had the highest TPC (45 mg GAE g^−1^ of yerba mate), but it was also the only mixture containing melissa, contributing to higher TPC. There was no statistically significant difference between TFC in WT and S extract and their two-component mixtures in the current study. The lowest TFC was determined in the *L* extract and its two- and three-component mixtures. Our findings were not supported by Studzińska-Sroka et al. [49], whose results of TPC in herbal tea blends correlated with a high content of flavonoids in the mixture of mulberry leaves and cinnamon bark (70:30) (2.23 mg QE g^−1^ herbal blend). The results were also different in research by Malongane et al. [48], where bush tea alone contained the lowest flavonoid content, but the flavonoid content increased in two-component mixtures.

### 3.2. Polyphenolic Characterization of Sage, Wild Thyme and Laurel Herbal Extract and Their Mixtures

A UPLC/MS-MS analysis was carried out to investigate the polyphenolic profile of the *L*, *WT* and *S* herbal extracts and their mixtures. The results (Table 3) showed 27 phenolic compounds consisting of flavanols, flavonols, flavones, and hydroxycinnamic and hydroxybenzoic acids.

Among flavanols, compounds **16** and **17** were identified by comparison with authentic standards such as catechin and epicatechin. Catechin and epicatechin were mainly found in the extract of *L* and *WT*, while in two-component and three-component mixtures, the highest amount was in *WTS31* and *WTSL211*. These flavanols were previously found in the herbal extracts of laurel [51,52] and wild thyme [53,54,55] in varying amounts.

Among the flavonols, compounds **2** and **15** were identified as rutin and myricetin compared with the authentic standards. The compounds **3**, **10**, **13** and **14** were characterized by a specific fragment ion at *m*/*z* 287 comparable with kaempferol. They were tentatively recognized due to specific fragment loss as kaempferol-3-rutinoside (rhamnose −146 amu; glucose −162 amu), kaempferol-3-*O*-hexoside (glucose −162 amu), kaempferol-3-*O*-deoxyhexoside (deoxyhexose −146 amu) and kaempferol-3-*O*-pentoside (pentose −132 amu) [33]. The compounds **6**, **9** and **12** were tentatively assigned due to the characteristic fragment ion at *m*/*z* 303 and specific loss of sugar moieties as quercetin-3-glucoside (glucose −162 amu), quercetin-3-rhamnoside (rhamnose −146 amu) and quercetin-3-pentoside (pentose −132 amu). The results of the current study showed that flavonols were the most abundant compounds in the extract of *S*, which was previously noted by Marchica et al. [56]. Flavonols, primarily kaempferol-3-*O*-hexoside and kaempferol-3-rutinoside, were also present in high amounts in the mixtures where *S* was dominant, but kaempferol-3-rutinoside was higher in most mixtures with *S* than in the one-component *S* extract. Its amount was dominant in the *WTS13* mixture, and kaempferol-3-*O*-hexoside was found in the highest concentration in the two-component *SL31* mixture. Rutin was dominant in all two-component *WTS* mixtures, while its presence was lower in their one-component extracts.

Among the flavones, compounds **18** and **19** were identified by comparison with authentic standards such as luteolin and apigenin. Apigenin was the dominant compound in the *S* extract and mixtures of *WT* with *S* and *L* (in the ratio of 2:1). In varying amounts, apigenin was also detected in all three-component mixtures. Luteolin was the most abundant in the *S* extract, but its amount was higher in the *SL31* mixture. All detected flavones have previously been found in the *WT*, *S* and *L* extracts [9,10,20,52,57,58]. Among the hydroxycinnamic acids, the compounds **20**, **21**, **24**, **25** and **27** were identified through comparison with authentic standards as rosmarinic, chlorogenic, ferulic, caffeic and *p*-caffeic acid. Caffeic acid dominated the *WT* and *S* extracts and their two-component mixtures. Except for caffeic acid, *p*-caffeic acid was also the dominant acid in the *WT* extract. The presence of these acids was remarkably higher in the *WT* extract than in any other *WT* mixtures. On the contrary, the presence of rosmarinic acid was higher in two-component *WT* mixtures than in the *WT* extract. Ferrulic acid was the most dominant in the *S* and two-component *S* mixtures. All detected hydroxycinnamic acids were found in previous studies of laurel [7,52,59,60], wild thyme [20,54,57,61] and sage extracts [8,19,56,61,62].

As for hydroxybenzoic acids, the compounds **22**, **23**, **26**, **28** and **29** were identified through comparison with authentic standards as 3,4-dihydrobenzoic acid hexoside, syringic, gallic, protocatechuic and *p*-hydroxybenzoic acids. Among them, protocatechuic acid was by far the most dominant. It was found in all extracts and mixtures, except for the *S* extract, where no presence of protocatechuic acid was detected. The amount of protocatechuic acid was the highest in the *L* extract, and its presence was reported in previous research [7,52].

### 3.3. Headspace Solid-Phase Microextraction (HS-SPME/GC-MS)

In the herbal extracts of *S*, *WT*, and *L*, volatile headspace compounds were isolated and analyzed by HS-SPME/GC-MS, and the results are shown in Table 4.

Monoterpenes were the most common isolated volatile organic compounds in the examined extracts. The oxygenated monoterpenes β-thujone (32.73%), α-thujone (17.99%), camphor (14.42%) and 1,8-cineole (13.08%) were the major constituents in the aqueous extract of S. These monoterpenes are well known for their antimicrobial, anti-inflammatory and antioxidant properties [63] and were also previously reported as the major constituents in sage hydrolate [12,64,65] and in much higher concentrations in a sage essential oil [66,67,68,69]. Additionally, Baydar et al. [12] compared distilled and extracted products of sage and determined that 1,8-cineole and camphor were present in greater quantities in the hydrolate in comparison to the essential oil, which can be explained by their better solubility in water. The essential oils containing thujones are believed to have potential neurotoxic and hepatotoxic effects that are not necessarily related only to thujone content but also to the presence of other components in the essential oils [66,70,71]. However, since thujones are less soluble in water, their minor presence in aqueous extracts could be associated with the mentioned toxic effects. However, it is still necessary to determine possible toxicity levels in herbal infusions because the trend of consuming them daily is growing [71,72]. Furthermore, the presence of thujones, camphor and 1,8-cineole also contribute to sensory features, providing minty and fresh odors and eucalyptus aromas, which is significant for functional beverages production [73,74].

The results obtained for the *WT* extract showed that the main volatile compound was carvacrol (19.24%), while thymoquinone, geraniol and thymol were present at concentrations of 11.3, 11.7 and 11.9%, respectively. Previously, the studies reported carvacrol and thymol as the most abundant compounds in wild thyme essential oil [75,76,77]. At the same time, thymol is also reported as the main compound of wild thyme hydrolate [78] and hydrolates of other *Thymus* species such as *Thymus mastichina* L. [79], *T. vulgaris* and *Thymus zygis* Loefl. ex L. [80]. Carvacrol and thymol are monoterpene phenols with powerful antiseptic, antibacterial, antioxidant, antifungal, anti-inflammatory and anticancer properties [81,82,83,84,85,86]. Furthermore, the current study results have shown the presence of geraniol and thymoquinone as the compounds found in lower concentrations but with similar biological properties as carvacrol and thymol [87,88,89,90]. The application of thymol or carvacrol in food products inhibits quality loss of the product, but their addition at high levels can affect the sensory properties. Therefore, special attention should be paid to sensory analysis [91], especially because thymol is the most active compound of the *WT* extract that contributes to the aromatic profile, providing a pungent and herbaceous aroma and characteristic thyme odor [14,92].

In the *L* aqueous extract, 1,8-cineole (34.61%), linalool (17.75%) and methyl eugenol (14.43%) were the major compounds. These results confirmed the compounds mentioned above as the main constituents of laurel hydrolate [64,93] and essential oil [94,95] in varying amounts. It is considered that methyl eugenol is a limiting factor that allows the use of laurel essential oil in food applications [96] and, along with linalool, exhibits antioxidant, antimicrobial, anti-inflammatory and anticancer properties [94,97,98,99,100,101,102] and various effects on the central nervous system [98,103,104]. These compounds are the primary compounds responsible for the aroma of laurel leaves [105]. Still, the low water solubility of linalool and methyl eugenol is a severe limit to their application in an aqueous environment [106,107].

Almost all of the previously mentioned compounds (1,8-cineole, linalool, β-thujone, α-thujone camphor and carvacrol) were present in lower abundance in the two- and three-component mixture headspace, depending on the ratio of the herbal extracts. It was predicted that two-component mixtures of *S* and *L* would have the highest amount of 1,8-cineole since the abundance of 1,8-cineole was the highest in their extracts, but the obtained results did not match our expectations. Although a modest abundance of 1,8-cineole was found in the *WT* extract, it was the most abundant headspace constituent in the *WTL13* mixture (29.32%). Linalool was also detected in two-component mixtures where *L* was dominant, primarily in *SL13* and *WTL13* (14.56 and 14.27%, respectively), whereas it was absent in three-component mixtures in concentrations higher than 10%. β-thujone and α-thujone are related to *S* mixtures, so the abundance of these molecules was higher in two-component mixtures where *S* predominated, for example, *SL31* and *WTS13* (30.72%; 30.55% for β-thujone and 18.03%; 17.52% for α-thujone). The percentage of α-thujone was slightly higher in the *SL31* mixture than in the pure extract of *S* (17.99%). In three-component mixtures, the abundance of these compounds is still high (23.36 and 12.73% in *WTSL121* for β- and α-thujone), but because of a lower amount of the *S* extract, these values were expectedly lower. Camphor and carvacrol are more specific for *S* and *WT*, whereas the presence of these molecules was not established in the *L* extract. However, carvacrol was still found in the mixtures of *L*, with the highest amount in *WTL31* (12.64%). On the other hand, the highest abundance of camphor was found in the *WTS13* mixture (11.66%), followed by the *SL31* mixture (10.89%).

Aside from the already described compounds, HS-SPME/GC-MS analysis showed the presence of other bioactives that also contributes to sensory properties, although they are represented in lower percentages. One of them is α-terpineol, the most commercially important monoterpene alcohol in the flavor industry with a floral, typically lilac odor [108]. Obtained results showed the presence of α-terpineol in the herbal extracts and mixtures, but it was mainly specific to *L* (6.39%) and mixtures where *L* dominates, such as *SL13* (5.23%), *WTL13* (4.9%) and *WTSL112* (3.24%). Borneol is another compound that occurs in lower abundance and can contribute to the aromatic profile. Borneol is a compound that provides a fragrant, spicy and cool flavor [109]. Analysis showed the presence of borneol in the extracts of *WT* and *S* (6.12%; 3.71%) and their two-component mixtures, where the highest percentage of borneol was found in the *WTS11* mixture (4.07%), followed by *WTSL221* (3.63%) in the three-component mixture. 1,4-Dihydroxy-2,5-di-tert-butylbenzene occurred in a lower percentage in the *WT* extract (7.66%) and a much higher percentage in the two-component *WTL31* and *WTS31* mixtures (18.51 and 16.75%).

### 3.4. Antioxidant Capacity

All polyphenols determined in the current study are known as bioactive molecules with health-promoting benefits, such as influencing several types of cancer, inflammation, autoimmune and neurodegenerative disease [110,111,112,113]. Still, they also have antioxidant properties, which are crucial in functional beverage development. The most frequent in vitro assays that have been used to evaluate antioxidant capacity are DPPH and ORAC. The ORAC assay is a fluorescence method that involves a hydrogen atom transfer mechanism. The DPPH assay is a spectrophotometric method based on a single electron-transfer reaction and hydrogen atom transfer [114]. The results of antioxidant capacity in herbal extracts of *S*, *WT*, and *L* and their two- and three-component mixtures determined by the DPPH and ORAC methods are presented in Table 2. Both methods showed a high antioxidant capacity for the *L* extract. Although two-component mixtures with the *L* extract had lower TPC and TFC content, they had a higher antioxidant capacity (DPPH and ORAC), as proven by both assays. These results can be explained by the high content of hydroxybenzoic acids in the *L* extract and potentially synergism with other compounds (such as flavonols and hydroxycinnamic acids) that contributed to antioxidant capacity due to their chemical structure and are determined to be present in high amounts in the current study. Electron donor groups of phenolic acids (phenolic hydroxyl and carboxylic acid groups) may reduce the dissociation energy of the phenolic hydroxyl bond, thus amplifying the ability to remove free radicals. Additionally, the increasing number of phenolic hydroxyl and methoxy groups in phenolic acids enhances antioxidant activity [115]. The number and configuration of hydroxyl groups in flavonoids and condensed tannins substantially influence antioxidant activity. The structures of flavonoids that possess catechol structure and hydroxyl groups at the 3’-, 4’-, and 5’-positions in the B-ring, and 2,3 double bond combined with a 3-OH and 4-oxo group in the C-ring, are considered to be associated with more significant antioxidant activity [116].

When comparing the antioxidant capacity of *S* and *WT* based on DPPH analysis, a slightly higher value was present in the *S* extracts (578.81 ± 5.19 μmol TE mL^−1^), followed by *WT* (544.13 ± 5.19 μmol TE mL^−1^). These results are consistent with previous research reported by Brezoiu et al. [61]. They confirmed more effective radical scavenging activity of *S* extracts (180.81–236.43 mg TE g^−1^ extract and 3.42–7.08 g TE 100 g^−1^ herbal) compared to the *WT* extracts (161.61–185.89 mg TE g^−1^ extract and 1.90–2.65 g TE 100 g^−1^ herbal) for the DPPH assay.

The results obtained using the ORAC assay also pointed to the *L* extract possessing the highest antioxidant capacity (1896.10 ± 8.77 μmol TE mL^−1^). Zheng and Wang [47] reported ORAC analysis of 27 culinary herbs and 12 medicinal herbs, including *L*, *S* and some other herbs from the genus *Thymus,* such as *T. vulgaris* and *Thymus × citriodorus*. *L* showed higher antioxidant capacity than *S* (31.70 ± 0.97 µmol g^−1^ of fresh weight and 13.28 ± 0.40 µmol g^−1^) and all *Thymus* species.

As far as we know, there is still no research about synergistic interactions between the *L*, *S* and *WT* extracts. Still, some studies have reported a synergic interaction between bioactive molecules in some other herbal extracts. Ydyrys et al. [117] reported synergism between the *S*, *WT* and ziziphora (*Ziziphora bungeana* Juz.) extracts with black or green tea. The mixtures resulted in an increased antioxidant effect. Studzińska-Sroka et al. [49] demonstrated the highest antioxidant capacity for an herbal extract made of mulberry leaves and a mixture of mulberry leaves and cinnamon bark. An increase in the number of herbal mixtures resulted in a decrease in the antioxidant capacity. Thus, it was the lowest in the four- and five-component mixtures. However, they reported high antioxidant capacity in a six-component mixture. The present results also demonstrated lower antioxidant capacity in three-component than in two-component mixtures or single herbal extracts. One explanation could be the interactions between the compounds present in the herbal mixtures that can create complexes and potentially decrease polyphenols’ electron donation capacity, thus reducing their antioxidant activity. Another possible explanation for lower antioxidant capacity in herbal mixtures is the dilution effect—a greater amount of herbals that participated in the combinations resulted in less bioactive molecules, which are important for antioxidant capacity. Some of these bioactive molecules are previously mentioned (flavonols and phenolic acids) as well as the volatile compound 1,8-cineole with strong antioxidant properties [118]. The presence of 1,8-cineole was highest in *L* and two-component mixtures with *L*. This may contribute to the higher antioxidant capacity of *L* and its mixtures, probably because of synergism with phenolic compounds. *L* contained a higher amount of hydroxybenzoic acids when compared to *S* and *WT*, in which hydroxycinnamic acid extracts were more present. It was previously reported that gallic acid, one of the acids whose amount was higher in *L* compared to *S* and *WT*, showed the highest DPPH scavenging activity in relation to hydroxycinnamic acids [119]. Gallic acid also indicated a synergistic interaction with protocatechuic acid, resulting in their higher antioxidant capacity [120,121]. High antioxidant capacity was also demonstrated in the synergistic action between gallic acid and caffeic acid [18], which was mainly found in the WT extract in the current study. It was expected that the *WTL* mixture should also provide high antioxidant capacity. A contribution to the antioxidant capacity in the two-component *WTL* mixture certainly was provided by the presence of catechins, which is related to their hydroxyl groups in their molecular structures [51,122]. There is a study about their synergistic antioxidant effect when supplemented with whole green tea extract [123] and synergism with protocatechuic acid in antibacterial and antioxidant activities [124,125]. Catechins also impact sensory properties, enhancing the bitterness and astringency of the beverage models [122]. For a better understanding and determination of the biopotential of herbal extracts, it is important to know the composition of individual phenolic and volatile compounds since not all groups contribute equally to antioxidant capacity.

## 4. Conclusions

Herbal extracts of sage, wild thyme and laurel showed great antioxidant capacity due to the rich composition of non-volatile (flavanols, flavonols, flavones, hydroxycinnamic and hydroxybenzoic acids) and volatile (mono- and sesquiterpenes) bioactive molecules. An aqueous extract of wild thyme showed the highest TPC content (2.79 ± 0.04 g L^−1^), and an extract of laurel had the highest antioxidant capacity (781.62 ± 5.19 μmol TE mL^−1^ in the DPPH assay and 1896.10 ± 8.77 μmol TE mL^−1^ in the ORAC assay). Two-component mixtures with wild thyme as a dominant extract had a greater TPC content than three-component mixtures. More effective antioxidant capacity was recorded in two-component than three-component mixtures where laurel dominates due to better synergistic interactions between bioactive molecules, primarily protocatechuic acid, gallic acid, caffeic acid, kaempferol-3-*O*-hexoside, 1,8-cineole, β-thujone and carvacrol. Therefore, two-component mixtures have promising potential in the production of functional beverages. Still, future studies should be conducted on their sensory analysis to be accepted by consumers.

## Figures and Tables

**Table 1:** Preparation of two- and three-component herbal extract mixtures.

Herbal Extract and Mixtures	Herbs	Ratios (*v/v*)	Label
One-component extract	*Wild thyme (WT)*		*WT*
	*Sage (S)*		*S*
	*Laurel (L)*		*L*
	*WT + S*	1:1	*WTS11*
	*WT + S*	1:3	*WTS13*
	*WT + S*	3:1	*WTS31*
	*WT + L*	1:1	*WTL11*
Two-component mixtures	*WT + L*	1:3	*WTL13*
	*WT + L*	3:1	*WTL31*
	*S + L*	1:1	*SL11*
	*S + L*	1:3	*SL13*
	*S + L*	3:1	SL31
	*WT + S + L*	1:1:1	*WTSL111*
	*WT + S + L*	1:2:1	*WTSL121*
	*WT + S + L*	1:1:2	*WTSL112*
Three-component mixtures	*WT + S + L*	1:2:2	*WTSL122*
	*WT + S + L*	2:1:1	*WTSL211*
	*WT + S + L*	2:2:1	*WTSL221*
	*WT + S + L*	2:1:2	*WTSL212*

**Table 2:** Content of total phenols, flavonoids, hydroxycinnamic acids, flavonols and antioxidant capacity of laurel, wild thyme and sage extracts and their two- and three-component mixtures.

	TPC (g L^−1^)	TFC (g L^−1^)	THCA (g L^−1^)	TFLC (g L^−1^)	DPPH (μmol TE mL^−1^)	ORAC (μmol TE mL^−1^)
	*p* < 0.01 ^†^	*p* < 0.01 ^†^	*p* = 0.25 ^‡^	*p* = 0.39 ^‡^	*p* < 0.01 ^†^	*p* < 0.01 ^†^
*Laurel (L)*	1.18 ± 0.04 ^a,b^	0.14 ± 0.2 ^a^	1.08 ± 0.21 ^a^	1.06 ± 0.21 ^a^	781.62 ± 5.19 ^j^	1896.10 ± 8.77 ^h,i^
*Wild thyme (WT)*	2.79 ± 0.04 ^j^	0.56 ± 0.2 ^f,g,h^	1.20 ± 0.21 ^a^	0.96 ± 0.21 ^a^	544.13 ± 5.19 ^f,g^	1734.74 ± 8.77 ^d^
*Sage (S)*	2.49 ± 0.04 ^i^	0.62 ± 0.2 ^g,h^	1.02 ± 0.21 ^a^	0.93 ± 0.21 ^a^	578.81 ± 5.19 ^h^	1459.32 ± 8.77 ^c^
*WTS11*	2.13 ± 0.04 ^f,g,h^	0.66 ± 0.2 ^h^	1.09 ± 0.21 ^a^	0.94 ± 0.21 ^a^	553.50 ± 5.19 ^g,h^	1744.08 ± 8.77 ^d,e^
*WTS13*	2.27 ± 0.04 ^g,h,i^	0.64 ± 0.2 ^h^	1.11 ± 0.21 ^a^	1.01 ± 0.21 ^a^	551.94 ± 5.19 ^g,h^	1785.72 ± 8.77 ^e,f^
*WTS31*	2.51 ± 0.04 ^i^	0.63 ± 0.2 ^g,h^	1.13 ± 0.21 ^a^	0.95 ± 0.21 ^a^	521.94 ± 5.19 ^d,e,f^	1305.37 ± 8.77 ^b^
*WTL11*	1.91 ± 0.04 ^e,f^	0.41 ± 0.2 ^c^	0.62 ± 0.21 ^a^	0.50 ± 0.21 ^a^	547.56 ± 5.19 ^f,g^	1755.68 ± 8.77 ^d,e^
*WTL13*	1.56 ± 0.04 ^c,d^	0.26 ± 0.2 ^b^	0.39 ± 0.21 ^a^	0.35 ± 0.21 ^a^	679.12 ± 5.19 ^i^	1913.38 ± 8.77 ^i^
*WTL31*	2.33 ± 0.04 ^h,i^	0.50 ± 0.2 ^c,d,e,f^	0.83 ± 0.21 ^a^	0.65 ± 0.21 ^a^	532.56 ± 5.19 ^e,f,g^	1769.66 ± 8.77 ^d,e,f^
*SL11*	1.79 ± 0.04 ^d,e^	0.44 ± 0.2 ^c,d^	0.58 ± 0.21 ^a^	0.53 ± 0.21 ^a^	469.44 ± 5.19 ^a,b^	1304.70 ± 8.77 ^b^
*SL13*	1.52 ± 0.04 ^c^	0.30 ± 0.2 ^b^	0.36 ± 0.21 ^a^	0.32 ± 0.21 ^a^	676.31 ± 5.19 ^i^	1849.74 ± 8.77 ^g,h^
*SL31*	2.04 ± 0.04 ^f,g^	0.55 ± 0.2 ^e,f,g,h^	0.76 ± 0.21 ^a^	0.69 ± 0.21 ^a^	506.94 ± 5.19 ^c,d,e^	1229.36 ± 8.77 ^a^
*WTSL111*	1.14 ± 0.04 ^a,b^	0.49 ± 0.2 ^c,d,e,f^	0.82 ± 0.21 ^a^	0.74 ± 0.21 ^a^	504.12 ± 5.19 ^c,d,e^	1256.65 ± 8.77 ^a,b^
*WTSL121*	1.20 ± 0.04 ^a,b^	0.56 ± 0.2 ^f,g,h^	0.81 ± 0.21 ^a^	0.70 ± 0.21 ^a^	539.44 ± 5.19 ^f,g^	1810.04 ± 8.77 ^f,g^
*WTSL112*	0.97 ± 0.04 ^a^	0.44 ± 0.2 ^c,d^	0.64 ± 0.21 ^a^	0.56 ± 0.21 ^a^	449.12 ± 5.19 ^a^	1245.13 ± 8.77 ^a^
*WTSL122*	0.99 ± 0.04 ^a^	0.47 ± 0.2 ^c,d,e,f^	0.71 ± 0.21 ^a^	0.64 ± 0.21 ^a^	506.31 ± 5.19 ^c,d,e^	1260.94 ± 8.77 ^a,b^
*WTSL211*	1.35 ± 0.04 ^b,c^	0.51 ± 0.2 ^d,e,f^	0.88 ± 0.21 ^a^	0.74 ± 0.21 ^a^	501.94 ± 5.19 ^c,d^	1232.79 ± 8.77 ^a^
*WTSL221*	1.45 ± 0.04 ^c^	0.53 ± 0.2 ^d,e,f,g^	0.85 ± 0.21 ^a^	0.74 ± 0.21 ^a^	490.69 ± 5.19 ^b,c^	1301.07 ± 8.77 ^b^
*WTSL212*	1.00 ± 0.04 ^a^	0.45 ± 0.2 ^c,d,e^	0.70 ± 0.21 ^a^	0.61 ± 0.21 ^a^	502.87 ± 5.19 ^c,d^	1217.64 ± 8.77 ^a^

TPC = total phenol content, TFC = total flavonoid content, THCA = total hydroxycinnamic acid content, TFLC = total flavonol content. Results are expressed as mean ± SD. ^†^ Statistically significant variable at *p* ≤ 0.05. ^‡^ Statistically insignificant variable at *p* ≥ 0.05. Values with different letters are statistically different at *p* ≤ 0.05.

**Table 3:** Polyphenolic characterization (g L^−1^ of the sample) of sage, wild thyme and laurel herbal extract and their mixtures (Table 1).

**Flavanols**						**Flavonols**								
**Compound**	**1**	**7**	**11**	**16**	**17**	**2**	**3**	**6**	**9**	**10**	**12**	**13**	**14**	**15**
**Tentative Identification**	**Procyandinin Trimer**	**Epigallocatechin Gallate**	**Epicatechin Gallate**	**Catechin**	**Epicatechin**	**Ruthin**	**Kaempferol-3-rutinoside**	**Quercetin-3-glucoside**	**Quercetin-3-rhamnoside**	**Kaempferol-3-*O*-hexoside**	**Quercetin-3-pentoside**	**Kaempferol-3-*O*-deoxyhexoside**	**Kaempferol-3-*O*-pentoside**	**Myricetin**
	*p* < 0.01 *	*p* < 0.01 *	*p* < 0.01 *	*p* < 0.01 *	*p* < 0.01 *	*p* < 0.01 *	*p* < 0.01 *	*p* < 0.01 *	*p* < 0.01 *	*p* < 0.01 *	*p* < 0.01 *	*p* < 0.01 *	*p* < 0.01 *	*p* < 0.01 *
*Laurel (L)*	1.21 ± 0.02 ^h^	0.02 ± 0.00 ^a,b^	0.10 ± 0.00 ^e,f^	1.43 ± 0.03 ^l^	1.26 ± 0.03 ^j^	0.70 ± 0.20 ^a^	1.10 ± 0.74 ^a^	2.88 ± 0.16 ^d,e,f^	3.91 ± 0.06 ^i^	2.53 ± 1.58 ^a^	1.42 ± 0.04 ^h^	0.03 ± 0.00 ^h,i,j^	0.72 ± 0.10 ^b^	0.22 ± 0.02 ^a,b,c^
*Wild thyme (WT)*	0.32 ± 0.02 ^e^	0.03 ± 0.00 ^a,b,c^	0.05 ± 0.00 ^a^	1.19 ± 0.03 ^i,j^	1.22 ± 0.03 ^j^	2.72 ± 0.20 ^b,c,d,e,f^	10.80 ± 0.74 ^e^	1.49 ± 0.16 ^a,b^	0.61 ± 0.06 ^b,c^	6.43 ± 1.58 ^a^	0.22 ± 0.04 ^a^	0.01 ± 0.00 ^b,c^	4.51 ± 0.10 ^i,j^	0.20 ± 0.02 ^a,b^
*Sage (S)*	0.10 ± 0.02 ^a,b,c^	0.05 ± 0.00 ^f,g^	0.10 ± 0.00 ^d,e,f^	0.36 ± 0.03 ^a^	0.31 ± 0.03 ^a^	4.24 ± 0.20 ^g,h,i^	23.49 ± 0.74 ^f,g,h^	2.95 ± 0.16 ^d,e,f^	0.20 ± 0.06 ^a^	137.04 ± 1.58 ^j^	0.05 ± 0.04 ^a^	0.03 ± 0.00 ^j,k^	0.12 ± 0.10 ^a^	1.06 ± 0.02 ^f^
*WTS11*	0.06 ± 0.02 ^a^	0.03 ± 0.00 ^b,c,d^	0.11 ± 0.00 ^f,g^	1.27 ± 0.03 ^j,k^	1.30 ± 0.03 ^j^	12.69 ± 0.20 ^k^	40.67 ± 0.74 ^j^	10.73 ± 0.16 ^h^	0.87 ± 0.06 ^c,d^	35.38 ± 1.58 ^d^	0.77 ± 0.04 ^c,d^	0.01 ± 0.00 ^a^	1.73 ± 0.10 ^d,e^	1.28 ± 0.02 ^g^
*WTS13*	0.07 ± 0.02 ^a,b^	0.08 ± 0.00 ^h^	0.11 ± 0.00 ^f,g^	1.18 ± 0.03 ^h,i,j^	1.22 ± 0.03 ^j^	13.21 ± 0.20 ^k^	65.41 ± 0.74 ^l^	12.72 ± 0.16 ^i^	0.75 ± 0.06 ^b,c,d^	45.74 ± 1.58 ^e,f^	0.74 ± 0.04 ^b,c,d^	0.02 ± 0.00 ^e,f,g^	1.04 ± 0.10 ^b,c^	1.69 ± 0.02 ^i^
*WTS31*	0.09 ± 0.02 ^a,b,c^	0.03 ± 0.00 ^c,d,e^	0.06 ± 0.00 ^b^	1.40 ± 0.03 ^k,l^	1.44 ± 0.03 ^k^	23.21 ± 0.20 ^l^	48.87 ± 0.74 ^k^	17.56 ± 0.16 ^j^	1.35 ± 0.06 ^f,g^	38.61 ± 1.58 ^d,e^	1.54 ± 0.04 ^h^	0.02 ± 0.00 ^d^	7.39 ± 0.10 ^k^	1.43 ± 0.02 ^h^
*WTL11*	0.17 ± 0.02 ^b,c,d^	0.03 ± 0.00 ^a,b,c^	0.08 ± 0.00 ^b,c,d^	0.70 ± 0.03 ^c,d^	0.80 ± 0.03 ^d,e^	1.97 ± 0.20 ^b,c^	2.71 ± 0.74 ^a,b^	3.09 ± 0.16 ^e,f^	1.98 ± 0.06 ^h^	35.20 ± 1.58 ^d^	1.52 ± 0.04 ^h^	0.03 ± 0.00 ^i,j,k^	5.05 ± 0.10 ^j^	1.25 ± 0.02 ^g^
*WTL13*	0.56 ± 0.02 ^g^	0.03 ± 0.00 ^b,c,d^	0.11 ± 0.00 ^f^	0.54 ± 0.03 ^b^	0.54 ± 0.03 ^b^	2.56 ± 0.20 ^b,c,d,e^	3.95 ± 0.74 ^a,b,c^	4.87 ± 0.16 ^g^	3.65 ± 0.06 ^i^	23.40 ± 1.58 ^b,c^	2.42 ± 0.04 ^j^	0.01 ± 0.00 ^a,b^	4.15 ± 0.10 ^h,i^	1.44 ± 0.02 ^h^
*WTL31*	0.11 ± 0.02 ^a,b,c^	0.04 ± 0.00 ^d,e^	0.08 ± 0.00 ^c,d,e^	0.97 ± 0.03 ^f,g^	1.03 ± 0.03 ^f,g,h^	5.19 ± 0.20 ^i,j^	5.56 ± 0.74 ^b,c,d^	3.61 ± 0.16 ^f^	2.07 ± 0.06 ^h^	56.45 ± 1.58 ^g,h^	1.51 ± 0.04 ^h^	0.02 ± 0.00 ^e^	2.65 ± 0.10 ^f,g^	0.22 ± 0.02 ^a,b,c^
*SL11*	0.09 ± 0.02 ^a,b,c^	0.03 ± 0.00 ^c,d^	0.11 ± 0.00 ^f,g^	0.66 ± 0.03 ^b,c^	0.70 ± 0.03 ^c,d^	1.77 ± 0.20 ^a,b^	8.69 ± 0.74 ^d,e^	1.88 ± 0.16 ^a,b,c^	1.35 ± 0.06 ^f,g^	55.52 ± 1.58 ^g,h^	0.87 ± 0.04 ^d,e^	0.01 ± 0.00 ^c^	0.90 ± 0.10 ^b^	0.28 ± 0.02 ^b,c^
*SL13*	0.23 ± 0.02 ^d,e^	0.05 ± 0.00 ^f,g^	0.09 ± 0.00 ^c,d,e^	0.56 ± 0.03 ^b,c^	0.63 ± 0.03 ^b,c^	1.73 ± 0.20 ^a,b^	7.16 ± 0.74 ^c,d,e^	3.26 ± 0.16 ^e,f^	3.81 ± 0.06 ^i^	45.81 ± 1.58 ^e,f^	1.84 ± 0.04 ^i^	0.03 ± 0.00 ^f,g,h^	1.58 ± 0.10 ^c,d^	0.20 ± 0.02 ^a,b^
*SL31*	0.13 ± 0.02 ^a,b,c,d^	0.02 ± 0.00 ^a^	0.13 ± 0.00 ^h,i^	0.35 ± 0.03 ^a^	0.31 ± 0.03 ^a^	4.70 ± 0.20 ^g,h,i^	22.80 ± 0.74 ^f,g,h^	2.55 ± 0.16 ^c,d,e^	1.08 ± 0.06 ^d,e,f^	140.19 ± 1.58 ^j^	1.05 ± 0.04 ^e,f^	0.03 ± 0.00 ^k^	1.01 ± 0.10 ^b^	0.60 ± 0.02 ^e^
*WTSL111*	0.06 ± 0.02 ^a^	0.03 ± 0.00 ^c,d,e^	0.08 ± 0.00 ^b,c^	1.06 ± 0.03 ^g,h,i^	1.06 ± 0.03 ^g,h,i^	2.90 ± 0.20 ^c,d,e,f^	20.05 ± 0.74 ^f^	0.96 ± 0.16 ^a^	0.60 ± 0.06 ^b,c^	47.46 ± 1.58 ^e,f,g^	0.51 ± 0.04 ^b^	0.01 ± 0.00 ^b,c^	2.16 ± 0.10 ^e,f^	0.22 ± 0.02 ^a,b,c^
*WTSL121*	0.19 ± 0.02 ^c,d^	0.03 ± 0.00 ^c,d,e^	0.07 ± 0.00 ^b,c^	1.04 ± 0.03 ^g^	1.16 ± 0.03 ^h,i,j^	4.89 ± 0.20 ^h,i^	45.51 ± 0.74 ^k^	3.04 ± 0.16 ^e,f^	1.45 ± 0.06 ^g^	103.56 ± 1.58 ^i^	1.32 ± 0.04 ^g,h^	0.03 ± 0.00 ^g,h,i^	3.17 ± 0.10 ^g^	0.34 ± 0.02 ^c,d^
*WTSL112*	0.43 ± 0.02 ^f^	0.05 ± 0.00 ^g^	0.18 ± 0.00 ^j^	0.88 ± 0.03 ^e,f^	0.86 ± 0.03 ^e^	6.09 ± 0.20 ^j^	28.39 ± 0.74 ^i^	5.30 ± 0.16 ^g^	2.26 ± 0.06 ^h^	30.49 ± 1.58 ^c,d^	2.56 ± 0.04 ^j^	0.01 ± 0.00 ^c^	4.64 ± 0.10 ^i,j^	0.44 ± 0.02 ^d^
*WTSL122*	1.21 ± 0.02 ^h^	0.04 ± 0.00 ^e^	0.15 ± 0.00 ^i^	0.95 ± 0.03 ^e,f,g^	1.01 ± 0.03 ^f,g^	3.11 ± 0.20 ^d,e,f^	24.30 ± 0.74 ^g,h,i^	1.26 ± 0.16 ^a,b^	0.89 ± 0.06 ^c,d,e^	58.77 ± 1.58 ^h^	0.67 ± 0.04 ^b,c,d^	0.02 ± 0.00 ^e,f^	1.93 ± 0.10 ^d,e^	0.44 ± 0.02 ^d^
*WTSL211*	0.06 ± 0.02 ^a^	0.04 ± 0.00 ^d,e^	0.13 ± 0.00 ^g,h^	1.27 ± 0.03 ^j,k^	1.17 ± 0.03 ^i,j^	2.20 ± 0.20 ^b,c,d^	25.68 ± 0.74 ^g,h,i^	2.08 ± 0.16 ^b,c,d^	1.22 ± 0.06 ^e,f,g^	60.88 ± 1.58 ^h^	0.90 ± 0.04 ^d,e^	0.01 ± 0.00 ^a,b,c^	4.12 ± 0.10 ^h,i^	0.25 ± 0.02 ^b,c^
*WTSL221*	0.06 ± 0.02 ^a^	0.05 ± 0.00 ^f^	0.10 ± 0.00 ^e,f^	1.04 ± 0.03 ^g,h^	1.01 ± 0.03 ^f,g,h^	3.60 ± 0.20 ^e,f,g^	26.12 ± 0.74 ^h,i^	1.15 ± 0.16 ^a^	0.47 ± 0.06 ^a,b^	20.88 ± 1.58 ^b^	0.53 ± 0.04 ^b,c^	0.02 ± 0.00 ^d^	2.53 ± 0.10 ^f^	0.13 ± 0.02 ^a^
*WTSL212*	0.09 ± 0.02 ^a,b,c^	0.04 ± 0.00 ^e^	0.11 ± 0.00 ^f^	0.82 ± 0.03 ^d,e^	0.89 ± 0.03 ^e,f^	3.83 ± 0.20 ^f,g,h^	21.52 ± 0.74 ^f,g^	2.43 ± 0.16 ^c,d,e^	1.22 ± 0.06 ^e,f,g^	52.83 ± 1.58 ^f,g,h^	1.17 ± 0.04 ^f,g^	0.02 ± 0.00 ^e^	3.93 ± 0.10 ^h^	0.21 ± 0.02 ^a,b^
**Flavones**				**Hydroxycinnamic Acids**					**Hydroxybenzoic Acids**					
**Compound**	**8**	**18**	**19**	**20**	**21**	**24**	**25**	**27**	**22**	**23**	**26**	**28**	**29**	
**Tentative Identification**	**Luteolin-6-C-Glucoside**	**Luteolin**	**Apigenin**	**Rosmarinic Acid**	**Chlorogenic Acid**	**Ferulic Acid**	**Caffeic Acid**	***p*-Caffeic Acid**	**3,4-Dihidrobenzoic Acid Hexoside**	**Syringic Acid**	**Gallic Acid**	**Protocatehuic Acid**	***p*-Hydroxybenzoic Acid**	
	*p* < 0.01 *	*p* < 0.01 *	*p* < 0.01 *	*p* < 0.01 *	*p* < 0.01 *	*p* < 0.01 *	*p* < 0.01 *	*p* < 0.01 *	*p* < 0.01 *	*p* < 0.01 *	*p* < 0.01 *	*p* < 0.01 *	*p* < 0.01 *	
*Laurel (L)*	1.11 ± 0.03 ^i^	1.09 ± 0.10 ^a^	0.94 ± 0.19 ^a^	0.14 ± 0.05 ^a^	0.19 ± 0.06 ^a^	1.23 ± 0.07 ^b^	2.34 ± 0.10 ^c,d^	2.79 ± 0.08 ^g,h,i^	0.50 ± 0.01 ^h^	0.14 ± 0.00 ^g,h,i^	6.06 ± 0.05 ^i^	92.80 ± 0.97 ^i^	5.38 ± 0.09 ^i^	
*Wild thyme (WT)*	0.08 ± 0.03 ^a^	5.07 ± 0.10 ^i,j^	6.28 ± 0.19 ^c,d,e^	1.45 ± 0.05 ^e^	4.54 ± 0.06 ^g^	0.30 ± 0.07 ^a^	17.72 ± 0.10 ^i^	13.27 ± 0.08 ^k^	0.27 ± 0.01 ^c,d,e^	0.15 ± 0.00 ^h,i^	0.25 ± 0.05 ^a,b^	13.55 ± 0.97 ^b^	3.80 ± 0.09 ^h^	
*Sage (S)*	0.37 ± 0.03 ^b^	2.21 ± 0.10 ^b^	3.33 ± 0.19 ^b^	2.00 ± 0.05 ^g^	0.09 ± 0.06 ^a^	4.47 ± 0.07 ^m^	9.76 ± 0.10 ^h^	1.86 ± 0.08 ^a,b^	0.34 ± 0.01 ^f,g^	0.01 ± 0.00 ^a^	0.13 ± 0.05 ^a^	0.00 ± 0.97 ^a^	1.43 ± 0.09 ^a^	
*WTS11*	0.37 ± 0.03 ^b^	3.96 ± 0.10 ^f,g^	8.15 ± 0.19 ^g,h^	4.34 ± 0.05 ^i^	3.45 ± 0.06 ^f^	4.44 ± 0.07 ^m^	7.67 ± 0.10 ^f^	2.69 ± 0.08 ^f,g,h^	0.32 ± 0.01 ^d,e,f^	0.16 ± 0.00 ^i^	0.18 ± 0.05 ^a^	26.53 ± 0.97 ^e,f^	2.20 ± 0.09 ^c,d^	
*WTS13*	0.35 ± 0.03 ^b^	5.15 ± 0.10 ^i,j^	9.72 ± 0.19 ^i^	3.97 ± 0.05 ^h^	2.21 ± 0.06 ^d^	5.94 ± 0.07 ^n^	8.27 ± 0.10 ^g^	2.13 ± 0.08 ^b,c,d,e^	0.38 ± 0.01 ^g^	0.02 ± 0.00 ^a,b^	0.16 ± 0.05 ^a^	15.80 ± 0.97 ^b^	1.61 ± 0.09 ^a,b^	
*WTS31*	0.85 ± 0.03 ^g,h^	4.31 ± 0.10 ^g,h^	13.08 ± 0.19 ^j^	4.75 ± 0.05 ^j^	5.99 ± 0.06 ^h^	3.16 ± 0.07 ^j,k,l^	7.11 ± 0.10 ^f^	3.16 ± 0.08 ^i^	0.50 ± 0.01 ^h^	0.19 ± 0.00 ^j^	0.18 ± 0.05 ^a^	40.14 ± 0.97 ^h^	2.97 ± 0.09 ^e,f,g^	
*WTL11*	0.98 ± 0.03 ^h,i^	2.97 ± 0.10 ^c,d^	9.78 ± 0.19 ^i^	1.56 ± 0.05 ^e,f^	2.88 ± 0.06 ^e^	1.12 ± 0.07 ^b^	2.32 ± 0.10 ^c,d^	2.38 ± 0.08 ^d,e,f,g^	0.25 ± 0.01 ^c,d^	0.14 ± 0.00 ^f,g,h,i^	0.82 ± 0.05 ^e,f^	27.08 ± 0.97 ^e,f^	2.25 ± 0.09 ^c,d^	
*WTL13*	1.54 ± 0.03 ^k^	4.63 ± 0.10 ^h,i^	6.77 ± 0.19 ^d,e,f^	1.80 ± 0.05 ^f,g^	1.46 ± 0.06 ^c^	1.49 ± 0.07 ^b,c^	1.15 ± 0.10 ^a^	1.94 ± 0.08 ^b,c^	0.23 ± 0.01 ^b,c^	0.13 ± 0.00 ^f,g^	2.01 ± 0.05 ^g^	33.25 ± 0.97 ^g^	3.08 ± 0.09 ^f,g^	
*WTL31*	0.83 ± 0.03 ^g,h^	3.33 ± 0.10 ^c,d,e^	13.88 ± 0.19 ^j^	1.45 ± 0.05 ^e^	3.36 ± 0.06 ^f^	1.51 ± 0.07 ^b,c^	2.68 ± 0.10 ^d,e^	2.35 ± 0.08 ^c,d,e,f^	0.26 ± 0.01 ^c,d^	0.12 ± 0.00 ^f^	0.40 ± 0.05 ^a,b,c^	26.65 ± 0.97 ^e,f^	2.40 ± 0.09 ^c,d^	
*SL11*	0.56 ± 0.03 ^d,e,f^	3.71 ± 0.10 ^e,f^	2.62 ± 0.19 ^b^	0.17 ± 0.05 ^a^	0.10 ± 0.06 ^a^	2.17 ± 0.07 ^e,f^	2.17 ± 0.10 ^c,d^	1.45 ± 0.08 ^a^	0.17 ± 0.01 ^a^	0.04 ± 0.00 ^b,c,d^	0.80 ± 0.05 ^d,e,f^	23.80 ± 0.97 ^e,f^	2.06 ± 0.09 ^b,c^	
*SL13*	1.33 ± 0.03 ^j^	3.41 ± 0.10 ^d,e,f^	2.44 ± 0.19 ^b^	0.31 ± 0.05 ^a,b,c^	0.06 ± 0.06 ^a^	2.49 ± 0.07 ^f,g,h^	1.35 ± 0.10 ^a,b^	4.78 ± 0.08 ^j^	0.23 ± 0.01 ^b,c^	0.04 ± 0.00 ^b,c,d^	3.42 ± 0.05 ^h^	42.95 ± 0.97 ^h^	4.98 ± 0.09 ^i^	
*SL31*	0.67 ± 0.03 ^e,f^	5.40 ± 0.10 ^j^	6.32 ± 0.19 ^c,d,e^	0.76 ± 0.05 ^d^	0.10 ± 0.06 ^a^	3.50 ± 0.07 ^l^	3.13 ± 0.10 ^e^	2.03 ± 0.08 ^b,c,d^	0.34 ± 0.01 ^f,g^	0.05 ± 0.00 ^d^	0.51 ± 0.05 ^b,c,d^	17.98 ± 0.97 ^b,c,d^	2.59 ± 0.09 ^d,e^	
*WTSL111*	0.44 ± 0.03 ^b,c,d^	2.92 ± 0.10 ^c,d^	5.69 ± 0.19 ^c,d^	0.22 ± 0.05 ^a,b^	0.32 ± 0.06 ^a^	1.69 ± 0.07 ^c,d^	2.33 ± 0.10 ^c,d^	2.38 ± 0.08 ^d,e,f,g^	0.32 ± 0.01 ^e,f^	0.03 ± 0.00 ^a,b,c^	0.30 ± 0.05 ^a,b,c^	15.77 ± 0.97 ^b^	2.10 ± 0.09 ^c^	
*WTSL121*	0.71 ± 0.03 ^f,g^	4.40 ± 0.10 ^g,h^	8.21 ± 0.19 ^g,h^	2.03 ± 0.05 ^g^	1.49 ± 0.06 ^c^	2.76 ± 0.07 ^h,i,j^	3.01 ± 0.10 ^e^	2.52 ± 0.08 ^e,f,g^	0.36 ± 0.01 ^f,g^	0.02 ± 0.00 ^a,b^	0.41 ± 0.05 ^a,b,c^	21.60 ± 0.97 ^c,d,e^	2.59 ± 0.09 ^d,e,f^	
*WTSL112*	1.55 ± 0.03 ^k^	3.43 ± 0.10 ^d,e,f^	6.54 ± 0.19 ^c,d,e,f^	1.53 ± 0.05 ^e,f^	2.14 ± 0.06 ^d^	3.36 ± 0.07 ^k,l^	2.59 ± 0.10 ^d,e^	1.94 ± 0.08 ^b,c^	0.32 ± 0.01 ^e,f^	0.31 ± 0.00 ^k^	1.01 ± 0.05 ^f^	27.87 ± 0.97 ^f,g^	3.28 ± 0.09 ^g^	
*WTSL122*	0.54 ± 0.03 ^c,d,e^	3.30 ± 0.10 ^c,d,e^	5.58 ± 0.19 ^c^	0.17 ± 0.05 ^a^	0.20 ± 0.06 ^a^	1.97 ± 0.07 ^d,e^	1.42 ± 0.10 ^a,b^	2.30 ± 0.08 ^c,d,e,f^	0.19 ± 0.01 ^a,b^	0.04 ± 0.00 ^b,c,d^	0.41 ± 0.05 ^a,b,c^	16.93 ± 0.97 ^b,c^	3.14 ± 0.09 ^g^	
*WTSL211*	0.65 ± 0.03 ^e,f^	7.69 ± 0.10 ^k^	8.54 ± 0.19 ^h^	0.58 ± 0.05 ^c,d^	1.60 ± 0.06 ^c^	2.71 ± 0.07 ^g,h,i^	1.79 ± 0.10 ^b,c^	2.64 ± 0.08 ^f,g,h^	0.24 ± 0.01 ^b,c,d^	0.09 ± 0.00 ^e^	0.29 ± 0.05 ^a,b,c^	22.96 ± 0.97 ^d,e,f^	2.38 ± 0.09 ^c,d^	
*WTSL221*	0.40 ± 0.03 ^b,c^	3.28 ± 0.10 ^c,d,e^	7.41 ± 0.19 ^f,g^	0.26 ± 0.05 ^a,b^	0.99 ± 0.06 ^b^	2.31 ± 0.07 ^e,f,g^	2.20 ± 0.10 ^c,d^	3.02 ± 0.08 ^h,i^	0.28 ± 0.01 ^d,e,f^	0.04 ± 0.00 ^c,d^	0.24 ± 0.05 ^a,b^	15.59 ± 0.97 ^b^	2.31 ± 0.09 ^c,d^	
*WTSL212*	0.85 ± 0.03 ^g,h^	2.80 ± 0.10 ^c^	6.97 ± 0.19 ^e,f^	0.47 ± 0.05 ^b,c^	1.97 ± 0.06 ^d^	3.01 ± 0.07 ^i,j,k^	1.42 ± 0.10 ^a,b^	2.60 ± 0.08 ^f,g,h^	0.34 ± 0.01 ^f,g^	0.13 ± 0.00 ^f,g,h^	0.57 ± 0.05 ^c,d,e^	33.17 ± 0.97 ^g^	2.05 ± 0.09 ^b,c^	

Results are expressed as mean ± SD. * Statistically significant variable at *p* ≤ 0.05. Values with different letters are statistically different at *p* ≤ 0.05.

**Table 4:** The headspace chemical composition of sage, wild thyme and laurel herbal extract and their mixtures (Table 1) as determined by HS-SPME/GC-MS analysis.

	**1,8-Cineole**	**Linalool**	**β-Thujone**	**α-Thujone**	**Camphor**	**Thymol**	**Carvacrol**	**Methyleugenol**	**Thymoquinone**	**Geraniol**	
*Laurel (L)*	34.61 ± 0.57	17.75 ± 0.53	-	-	-	-	-	14.43 ± 0.30	-	-	
*Wild thyme (WT)*	-	-	1.19 ± 0.06	-	2.87 ± 0.06	11.9 ± 0.28	19.24 ± 0.18	-	11.3 ± 0.21	11.7 ± 0.42	
*Sage (S)*	13.08 ± 0.09	1.48 ± 0.02	32.73 ± 0.53	17.99 ± 1.04	14.42 ± 0.29	-	-	-	-	-	
*SL11*	20.94 ± 1.20	9.41 ± 0.22	21.39 ± 1.70	11.34 ± 0.24	9.89 ± 0.63	-	-	6.22 ± 0.16	-	-	
*SL13*	24.58 ± 1.05	14.56 ± 0.43	12.36 ± 0.34	6.44 ± 0.24	6.21 ± 0.15	-	-	10 ± 0.14	-	-	
*SL31*	17.73 ± 0.62	3.98 ± 0.07	30.72 ± 0.35	18.03 ± 0.33	10.89 ± 0.69	-	-	2.22 ± 0.15	-	-	
*WTL11*	19.72 ± 1.32	10.16 ± 0.68	-	-	1.37 ± 0.26	5.95 ± 0.25	10.08 ± 0.40	6.62 ± 0.43	1.75 ± 0.07	5.28 ± 0.20	
*WTL13*	29.32 ± 0.93	14.27 ± 0.83	-	-	-	2.61 ± 0.08	4.58 ± 0.27	9.84 ± 0.98	-	2.35 ± 0.05	
*WTL31*	10.45 ± 0.42	6.56 ± 0.52	-	-	1.87 ± 0.15	7.62 ± 0.74	12.64 ± 0.77	2.87 ± 0.40	2.78 ± 0.13	6.66 ± 0.46	
*WTS11*	9.00 ± 0.09	2.03 ± 0.02	21.73 ± 0.93	11.28 ± 0.42	10.57 ± 0.75	4.37 ± 0.12	7.45 ± 0.25	-	2.05 ± 0.07	3.52 ± 0.10	
*WTS13*	11.68 ± 1.11	1.43 ± 0.16	30.55 ± 1.64	17.52 ± 1.64	11.66 ± 0.80	2.16 ± 0.11	3.67 ± 0.18	-	-	1.55 ± 0.04	
*WTS31*	5.06 ± 0.15	2.31 ± 0.08	13.78 ± 1.79	7.54 ± 0.38	6.34 ± 0.56	7.1 ± 0.18	11.74 ± 1.01	-	2.44 ± 0.10	5.68 ± 0.18	
*WTSL111*	16.23 ± 0.73	5.77 ± 0.47	18.09 ± 0.70	10.1 ± 0.49	7.05 ± 0.60	3.22 ± 0.16	5.53 ± 0.37	3.95 ± 0.59	-	2.4 ± 0.10	
*WTSL121*	15.51 ± 1.08	4.23 ± 0.16	23.36 ± 1.27	12.73 ± 1.08	9.00 ± 0.35	2.13 ± 0.09	3.75 ± 0.53	2.41 ± 0.22	-	1.54 ± 0.05	
*WTSL112*	23.29 ± 1.62	8.92 ± 0.65	13.66 ± 0.88	6.83 ± 0.58	6.35 ± 0.65	2.14 ± 0.10	3.87 ± 0.54	5.87 ± 0.54	-	1.49 ± 0.05	
*WTSL122*	18.85 ± 1.24	6.48 ± 0.33	20.79 ± 1.41	11.71 ± 1.13	7.8 ± 0.49	1.88 ± 0.25	3.27 ± 0.12	4.24 ± 0.09	-	1.32 ± 0.02	
*WTSL211*	11.66 ± 1.10	5.37 ± 0.20	13.41 ± 1.10	7.22 ± 0.25	6.53 ± 0.11	4.99 ± 0.05	8.33 ± 0.15	2.52 ± 0.10	1.31 ± 0.05	3.71 ± 0.07	
*WTSL221*	15.84 ± 1.82	4.75 ± 0.31	20.11 ± 1.98	10.34 ± 1.01	9.3 ± 0.45	3.67 ± 0.05	6.2 ± 0.10	2.07 ± 0.10	-	2.89 ± 0.02	
*WTSL212*	17.73 ± 1.10	7.6 ± 0.20	11.96 ± 0.98	6.52 ± 0.45	5.14 ± 0.36	4.22 ± 0.15	7.18 ± 0.22	4.83 ± 0.11	-	3.26 ± 0.10	
	**Eugenol**	**Borneol**	**4-Terpineol**	**α-Terpineol**	**α-Terpinyl acetate**	**Oct-1-en-3-ol**	**(*E*)-Citral**	**1,4-Dihydroxy-2,5-di-tert-butylbenzene**	**γ-Terpinene**	**Veridiflorol**	**Menthol**
*Laurel (L)*	4.13 ± 0.10	-	4.81 ± 0.15	6.39 ± 0.20	7.54 ± 0.33	-	-	-	-	-	-
*Wild thyme (WT)*	-	6.12 ± 0.21	6.25 ± 0.34	1.12 ± 0.10	-	2.29 ± 0.12	-	7.66 ± 0.98	2.29 ± 0.08	-	-
*Sage (S)*	-	3.71 ± 0.12	1.55 ± 0.20	-	-	-	-	-	-	1.63 ± 0.10	-
*SL11*	1.66 ± 0.05	2.77 ± 0.01	3.12 ± 0.04	3.18 ± 0.08	2.22 ± 0.09	-	-	-	-	-	-
*SL13*	2.63 ± 0.06	2.1 ± 0.07	4.34 ± 0.10	5.23 ± 0.15	3.6 ± 0.20	-	-	-	-	-	-
*SL31*	-	2.59 ± 0.20	1.81 ± 0.17	1.2 ± 0.05	1.42 ± 0.09	-	-	-	-	-	-
*WTL11*	1.99 ± 0.10	3.01 ± 0.17	5.28 ± 0.11	3.38 ± 0.06	4.19 ± 0.08	1.18 ± 0.05	-	8.73 ± 0.58	-	-	-
*WTL13*	2.67 ± 0.15	1.85 ± 0.07	5.21 ± 0.18	4.9 ± 0.20	6.87 ± 0.87	-	-	3.47 ± 0.10	-	-	-
*WTL31*	1.58 ± 0.04	4.03 ± 0.15	5.52 ± 0.25	2.13 ± 0.08	2.03 ± 0.05	1.55 ± 0.03	1.55 ± 0.02	18.51 ± 1.89	1.4 ± 0.02	-	-
*WTS11*	-	4.07 ± 0.02	3.05 ± 0.08	-	-	1.21 ± 0.05	1.28 ± 0.06	10.46 ± 1.20	-	-	-
*WTS13*	-	3.26 ± 0.15	2.06 ± 0.05	-	-	-	-	4.96 ± 0.29	-	-	-
*WTS31*	-	4.03 ± 0.15	3.88 ± 0.12	-	-	1.43 ± 0.05	1.62 ± 0.06	16.75 ± 1.88	1.01 ± 0.05	-	-
*WTSL111*	1.52 ± 0.10	2.78 ± 0.08	-	1.95 ± 0.05	2.23 ± 0.22	-	-	6.77 ± 0.20	-	-	3.27 ± 0.11
*WTSL121*	1.08 ± 0.05	3.03 ± 0.05	2.44 ± 0.58	1.37 ± 0.09	1.48 ± 0.15	-	-	6.7 ± 0.33	-	-	-
*WTSL112*	2.01 ± 0.07	2.71 ± 0.06	4.03 ± 0.10	3.24 ± 0.08	1.78 ± 0.16	-	-	4.82 ± 0.14	-	-	-
*WTSL122*	1.35 ± 0.05	2.57 ± 0.08	3.04 ± 0.10	2.15 ± 0.03	2.62 ± 0.19	-	-	2.88 ± 0.05	-	-	-
*WTSL211*	1.39 ± 0.04	3.61 ± 0.10	3.93 ± 0.23	1.82 ± 0.05	1.31 ± 0.20	1.19 ± 0.07	1.81 ± 0.10	12.48 ± 1.99	-	-	-
*WTSL221*	1.34 ± 0.04	3.63 ± 0.07	3.74 ± 0.11	1.62 ± 0.05	-	1.08 ± 0.05	-	2.1 ± 0.08	-	-	-
*WTSL212*	1.92 ± 0.02	2.97 ± 0.05	4.08 ± 0.25	2.61 ± 0.10	2.76 ± 0.11	-	-	5.5 ± 0.18	1.01 ± 0.05	-	-

Results are expressed as percentage composition as mean ± SD.

## Data Availability

The data presented in this study are available in this manuscript.
